# Factors Influencing the Variation of Plants’ Cardinal Temperature: A Case Study in Iran

**DOI:** 10.3390/plants13202848

**Published:** 2024-10-11

**Authors:** Sima Sohrabi, Javid Gherekhloo, Saeid Hassanpour-bourkheili, Afshin Soltani, Jose L. Gonzalez-Andujar

**Affiliations:** 1 Department of Agronomy, Ferdowsi University of Mashhad, Iran and Leader of Iranian Invasive Plants Working Group, Gorgan 4917739001, Iran; 2 Department of Agronomy, Gorgan University of Agricultural Sciences and Natural Resources, Gorgan 4913815739, Iran; gherekhloo@gau.ac.ir (J.G.); s.hassanpour.b@gmail.com (S.H.-b.); afshin.soltani@gmail.com (A.S.); 3 Instituto de Agricultura Sostenible (IAS-CSIC), 14004 Cordoba, Spain; andujar@csic.es

**Keywords:** alien plants, base temperature, establishment, germination, life cycle

## Abstract

The establishment and spread of plants in their native or alien geographical ranges are determined by their germination. This study investigated the impact of different factors on variations in cardinal temperatures. We used the lm procedure and measured the effect size by the Eta-square approach to find the association of different factors (species, ecotypes, origin (native/alien), year, and life cycle) with the cardinal temperatures of 31 species. Our results showed that the base, optimum, and maximum temperatures responded differently to these factors. The base temperature was less impacted by ecotypes compared with the optimum and maximum temperatures, whereas the species had a higher impact on the variation in the base temperature. The effect of the origin of weedy plants on the base temperature was higher than the optimum and maximum temperatures. The effect of the year on the optimum temperature was more prominent than that on the base and maximum temperatures. The results confirmed that weedy alien plants preferred high and narrow ranges of base, optimum, and maximum temperatures and probably will be more problematic in summer crops. The results indicate that alien plants can benefit from warmer conditions in invaded areas at the germination stage. These findings lay the foundation for further studies to elucidate which factors are more important.

## 1. Introduction

Seed germination is a critically important stage of life in plants and the consequent success or failure of the plant’s establishment heavily depends on this process [1,2,3]. The timing of germination also plays a significant role in the inter- and intra-specific competition between plants [4,5]. The variation in seed response between and within populations is one of the key factors responsible for the establishment and persistence of alien plant species [6,7,8]. The invasiveness and pre-adapting ability of an alien plant are associated with certain attributes, including rapid and prolific germination, rapid growth and high fecundity, and great environmental tolerance [9,10,11]. Despite increased scientific efforts to study biological invasions of certain plant species, there is still a lack of adequate understanding of how alien vs. native plant species respond to environmental cues in different regions. The responses of alien and native species will vary depending on a number of factors, including the habitat, species, environmental parameters, distribution range, invasion status, and the intensity of management measures [12,13,14]. It is more probable that alien species with wider distribution ranges have a higher chance of adapting to various conditions compared to alien plants with a limited distribution [15]. Therefore, it is essential to implement rigorous management measures to prevent alien plants from adapting to environmental stresses [16,17]. Environmental factors are mentioned as one of the main barriers to invasion by alien plants [18]. The decision on which alien species should be prioritized for management will depend on the potential for an alien species to invade and its negative impact on the region’s agricultural diversity and biodiversity [18,19].

Each plant species has its specific range of cardinal temperatures with base (T_b_), optimum (T_opt_), and maximum (T_max_) temperatures that determine the geographical limits for growth [20]. The maximum growth and development rate occurs around the optimum temperature (T_opt_) range [21,22,23]. The base and maximum temperatures are the lowest and highest temperatures, respectively, at which a plant is able to grow [24] and germinate [25]. Cardinal temperatures can be estimated from data on plant development—which is primarily a temperature-dependent process—by conducting germination tests under experimental conditions within a range of constant temperatures. Various non-linear functions are applied to describe specific ranges of cardinal temperatures, which vary in terms of simplicity and realistic description [26]. Among these functions are Dent, segmented, and beta models, which have been frequently used to estimate the cardinal temperatures of germination in numerous crops and weeds such as safflower (*Carthamus tinctorius* L.) [27]; Asian spiderflower (*Cleome viscosa* L.) [28]; purple nutsedge (*Cyperus rotundus* L.) [29]; sea barley (*Hordeum marinum* Huds.) [30]; etc.

The response of germination to temperature depends on various factors such as the plant’s species, variety, growth environment, or origin [26,31,32]. Understanding how alien plants respond to temperature is crucial for detecting their competitive ability and their response to climate change [33], which may differ from that of native species [34,35]. A substantial body of research has demonstrated that the cardinal temperature exhibits considerable variation across different populations. However, there is currently a paucity of information regarding the full extent of this variation. Moreover, no research has been conducted to ascertain the most influential factors responsible for fluctuations within the cardinal temperatures of germination. Consequently, there is a need to determine the impact of various factors on the three cardinal temperature components. Thus, the objective of this study was to evaluate the effect of the species, ecotypes, origin (native/alien), year, and life cycle of plant species on the variation in the base, optimum, and maximum seed temperatures.

## 2. Results

### 2.1. Description of Dataset

**Figure 1:**
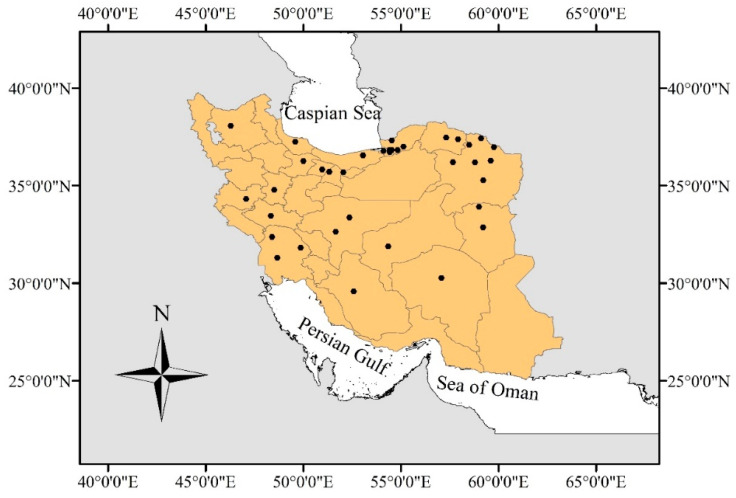
The dots show distribution of the examined populations in Iran.

From 84 selected records, 46 ecotypes and 31 species were obtained, which belonged to 14 families. Most of the records were associated with the Poaceae, Asteraceae, Brassicaceae, and Plantaginaceae families. Of the 31 different species studied, 8 species were alien (19 records) and belonged to five families (Supplementary Data). The species studied were distributed almost all over Iran, with most of them occurring in the northern and northeastern parts of the country (Figure 1).

### 2.2. The lm Procedure Output

**Figure 2:**
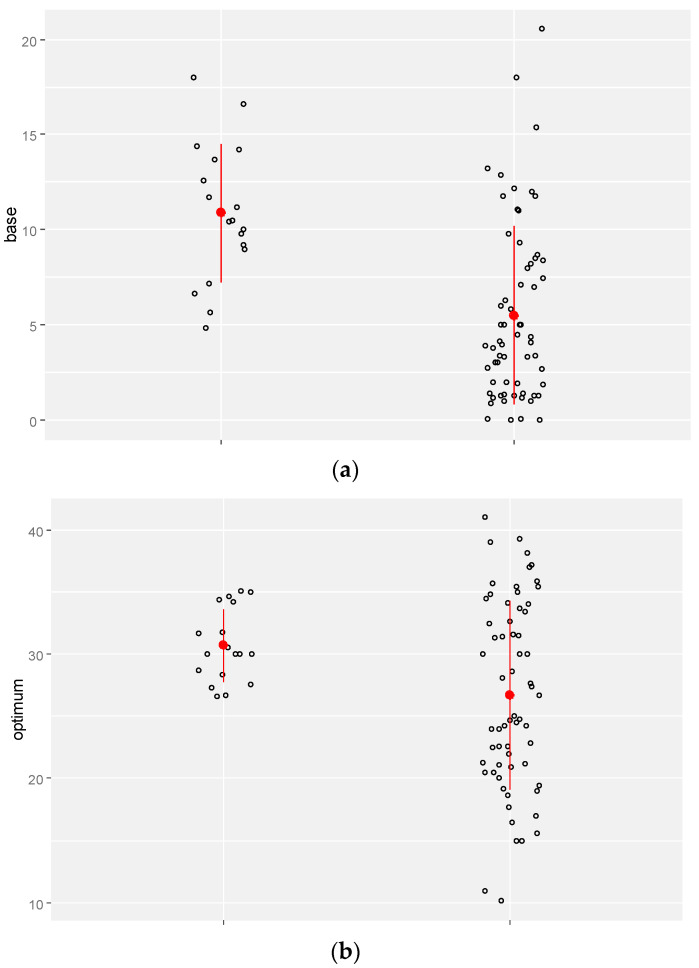

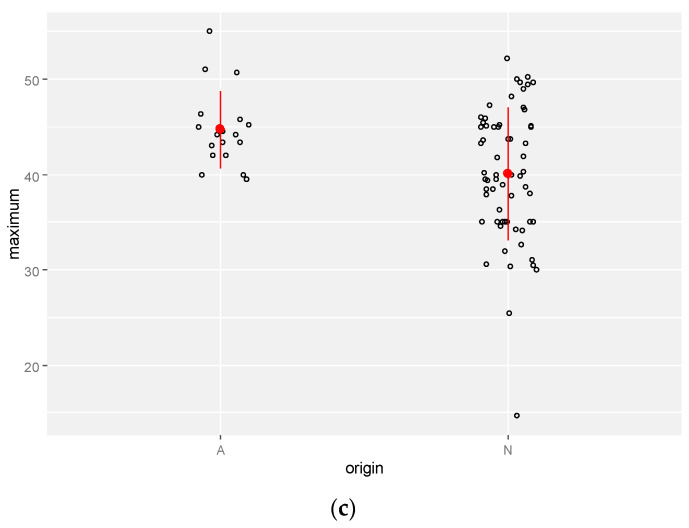
The effect of origin on the variation in the base (**a**) (mean = 5.51 for native plants (N) and 10.6 for alien plants; *p*-value: <0.001), optimum (**b**) (mean = 26.7 for native plants and 30.9 for alien plants; *p*-value: 0.003), and maximum (**c**) temperature (mean = 40 for native plants and 44.9 for alien plants; *p*-value: <0.001).

The lm procedure (a two-way ANOVA linear model) showed that some factors are more important than others with regard to the variation in cardinal temperatures. Being of native or alien origin had a significant impact on the variation in base, optimum, and maximum temperatures. All the factors, apart from the life cycle, significantly affected the base and optimum temperatures. Our results revealed that the base temperature is less influenced by ecotypes than by the optimum and maximum temperatures. The maximum temperature was not affected by year despite the base and optimum temperatures (Table 1). Alien species had higher base temperatures (mean = 10.6 °C) than the native ones (mean = 5.51 °C), and the average optimum and maximum temperatures were around 31 and 45 °C, respectively. Alien plants were also subject to narrow optimum and maximum temperatures in comparison with the native species (Figure 2).

### 2.3. Effect Size of Factors

**Figure 3:**
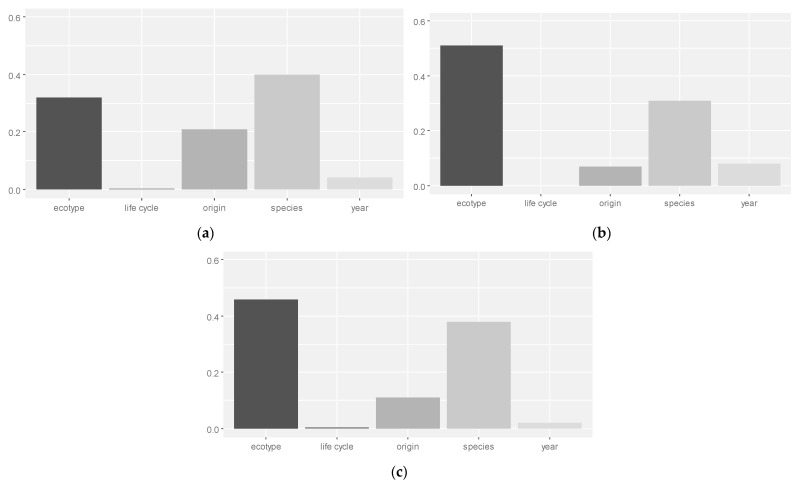
Relative effect sizes (Eta-squared) for the base (**a**), optimum (**b**), and maximum (**c**) temperatures by different factors.

The effect size in ANOVA measured the degree of association between the effect and the dependent variable. The interaction between the species and base, optimum, and maximum temperatures accounted for 40, 31, and 38% of the total variability, respectively. The effect of ecotypes on the variation in the base, optimum, and maximum temperatures explained 32, 51, and 46% of the total variability, respectively. Being native or alien had the most effect on the base temperature (21%), whereas its effect on the optimum and maximum temperatures was around 7 to 9%, respectively. The effect of the year of study on the optimum temperature was higher than that on the base and maximum temperatures (Figure 3). In this study, the base and maximum temperatures were more affected by the species type and not by the ecotypes. The latter had a greater effect on the variation in the optimum temperature. In general, the year and life cycle had a lesser influence on the variation in cardinal temperature (Figure 3).

## 3. Discussion

The species most represented in this study was related to two families with the largest number of plant species. Asteraceae and Poaceae contain the highest number of weedy species of all the flora of many other countries (https://powo.science.kew.org). Our result showed that the base temperature was affected by all the factors. Other studies have reported positive correlations between the base temperature and other germination traits [36]. Plant species can alter the base, optimum, and maximum temperature to cope with unpredictable conditions, in harmony with efficient adaptation strategies [37]. The genetic diversity of species, biodiversity level of the region, seed age, and climate conditions of the maternal plant have an effective role in their germination time and in the consequences of shaping a community assembly [38,39,40,41]. Species that detect and respond more quickly to environmental changes at ecological and genetic levels through adaptation are expected to have a selective advantage over species that respond more slowly [3]. Plant species’ response to environmental conditions changes over time and, being more acknowledged for the range of their response changes, will be necessary to predict their distribution and impact on different ecosystem levels [42,43,44]. The duration of the growing season and seed size had a considerable impact on the optimum temperature of *Nigella sativa* L. [45] and *Trigonella foenum-graecum* L. [32]. The effect of the year on the variation in cardinal temperatures can be attributed to the effect of the region’s annual rainfall, temperature, and amount of sunshine. Plant species that grow under optimum conditions will have a larger seed size and germinate faster, which influences cardinal temperatures [46,47,48]. The seed mass and germination were improved under a favorable habitat´s quality in different populations of *Scorzonera hispanica* L. [49]. The tolerance ranges (i.e., ecological valencies) to various environmental factors from the natural selection of plants will be important to predict their response in different locations, especially in introduced plants and their subsequent distribution [15,50]. More studies about different populations’ responses will be helpful to draw accurate conclusions about plants’ performance.

The wider base, optimum, and maximum temperature range in native species can be attributed to their greater genetic diversity in their native area. The lesser trait similarity between native and alien plants could lead to significant impacts of alien plants on the communities that they invade [51,52]. Alien plants had a higher base temperature than native plants, which suggests that growing in new regions with warmer temperatures may be more beneficial for alien species. Some alien plants are more competitive under climate change conditions due to their rapid establishment and growth in warmer temperatures [53]. In general, the variation in the cardinal temperature of native plants is higher than that of alien ones. The future flora of plant communities could be modified by the different responses of alien and native species to their germination stage [33,35]. Accordingly, Trotta et al. [33] reported that warmer temperatures favor the germination of alien plants rather than that of native species, so they may be more prone to emerge in summer crops as weeds. In Iran, 50% of alien plants grow in agricultural habitats, so that awareness of their response could help to develop robust management tactics [54]. Widespread invasive plants have been able to sense the changes in the climate and respond to them more rapidly via plastic and/or adaptive changes. Therefore, invasive species are predicted to have an advantage over slow-responding plants [55,56,57]. The prolific growth and worldwide distribution of *Amaranthus retroflexus* L. is related to its high invasion potential [6]. As the establishment and spread of plant species in their native or alien geographic areas are determined by germination as a key process and mechanism [58], finding out the timing of germination will be essential to estimate the growth, maturity, and seed production of the plants [59]. The increasing pressure from biological invasions on ecosystems, intensified by the effects of climate change, requires the swift development of robust and effective management strategies to control invasive species [60,61]. Invasive species, particularly weeds, represent a significant risk to agricultural productivity, biodiversity, and ecosystem services. It is therefore essential to adopt a comprehensive and adaptive approach to weed management, integrating multiple control techniques [62]. Preventive management is a vital tool in reducing the risk of new invasive species introductions, as well as in mitigating further detrimental impacts once they are established [63]. Preventative measures may include the introduction of stricter phytosanitary regulations, early detection and rapid response systems, and the use of certified weed-free seeds and planting materials. Furthermore, public awareness campaigns and education on the risks associated with invasive species are essential for reducing the likelihood of unintentional introductions through human activities.

New weed control tools are being developed that focus on species-specific characteristics and the ecological dynamics of invasion. These tools are particularly valuable for addressing alien species in the early “introduction” phase of invasion, where containment and localized eradication efforts are more feasible [64,65]. For example, early intervention techniques can prevent invasive weeds from becoming established and spreading across larger areas, thereby reducing long-term management costs and minimizing ecosystem disruption.

Control practices targeting weed seedbanks can be highly effective in limiting the persistence and spread of invasive species. One such method involves manipulating soil conditions, such as by lowering soil temperature, to delay seed germination. By extending the dormancy period, seeds are exposed to a longer period of vulnerability, increasing the likelihood of predation by natural enemies such as ants, beetles, and other seed predators [66]. This strategy not only increases seed mortality but also reduces the overall seedbank density, thereby limiting future weed infestations. Another successful approach is the use of weed emergence models [67]. These models are based on environmental factors, such as soil temperature, which influence weed seed dormancy and germination. Gaining insight into the factors influencing variations in cardinal temperatures allows the creation of more precise models and the implementation of timely control interventions, such as herbicide application or mechanical control. This significantly improves the efficiency of invasive species management. By accurately identifying the ideal environmental conditions for germination and growth, control measures can be better timed, ensuring maximum effectiveness and reducing the impact of invasive species on ecosystems and crops.

The effect size result demonstrated the strength of the relationship between plant origin and base temperature. Likewise, the ecotypes influenced the optimum temperature and the species influenced the base temperature. Effect sizes are the most important outcome of empirical studies and can show the magnitude of the reported effects [68,69]. Differential associations of factors in plant response to water stress were represented by effect size [70]. The current evaluations of the variation in cardinal temperature have focused on germination changes; however, increases in minimum temperature may be more significant in their effect on growth and phenology [71]. These findings could be further explored with different settings and more species in different communities and countries, adding depth to our understanding of the alien plant’s response to temperature.

We hope the aforementioned alien weeds garner significant attention from policymakers and prompt the necessary management actions in Iran, particularly given their advantage in thriving under warmer temperatures. Climate change is creating increasingly favorable conditions for these invasive species, allowing them to expand their range, outcompete native flora, and disrupt agricultural systems. The rise in temperatures not only accelerates the growth and reproduction of these weeds but also extends their growing seasons, making them more resilient to traditional control methods.

## 4. Materials and Methods

### 4.1. Data Collection

The database employed in this study was taken from peer-reviewed publications in the English and Persian languages (Supplementary Data) via the Web of Science, Google Scholar, Iranian journals, and congress proceedings. The search encompassed literature related to Iranian plant species (both native and alien) and cardinal temperature from 2020 to 2024, using the following terms: “cardinal temperature” or “weed germination”, or “plant germination” or “germination temperature” or “germination range” or “base temperature” or “optimum temperature”. The following criteria were applied for this investigation: (1) the outputs of the segmented model, including base, optimum, and maximum temperature, (2) the year (of seed collection), (3) the location of seed collection, (4) the origin of species selected (native or alien). The criteria mentioned above were extracted when there were at least two studies on the selected species (Table 2). We screened 84 records (31 different species) by using the above criteria (Supplementary Data). The determination of the origin (native or alien) and nomenclature of species were based on the POWO (Plants of the World Online) database (https://powo.science.kew.org). The location of the study was used to determine the ecotype as a population (or subspecies or race) that is adapted to local environmental conditions. We used the segmented model for the greater availability of information on the species in Iran [72]. A list of the species studied in this paper is presented in Table 2.

### 4.2. Statistical Analyses

The statistical analysis involved the linear model (lm) procedure and the calculation of effect size using Eta-squared (η^2^). We employed two-way analysis of variance (ANOVA) at a 0.05 significance level to assess the differences among factors concerning the three key temperature parameters. In the linear model, we focused on explaining the variance attributed to each model term, which facilitated the prediction and elucidation of variability among the factors analyzed through ANOVA.

To quantify the proportion of total variance in the dependent variable associated with the factors, we calculated effect sizes for the ANOVAs. The effect size reflects the strength of the association between the factors and the dependent variable, measured specifically by Eta-squared (η^2^), defined as η^2^ = SSeffect/SStotal. This calculation was implemented using the eta_squared function from the effectsize library [69]. For each factor, we utilized the model to examine interactions with reference to the base, optimum, and maximum temperatures. Data processing and statistical analyses were conducted using R version 4.3.0 beta (R Core Team), with the effect size and ggplot2 (v. 3.4.2) packages employed for enhanced data visualization.

## 5. Conclusions

This study provided the first comparison of the effect of different factors on three components of cardinal temperature and showed that the factors examined had significant effects apart from the life cycle of the species. Variations in base temperature were more affected by the plant’s species, while optimum temperature was more influenced by its ecotype. Our results also demonstrated that the base temperature was more affected by the plant’s origin, so that alien plants preferred a higher and narrower range of base, optimum, and maximum temperatures than the native ones. From this evidence, it was elucidated that alien plants can benefit more under warmer conditions in invaded areas and may be more problematic in summer crops. These findings lay the foundation for carrying out subsequent studies with broader species ranges of native and alien plants. The comparison of the response of these plants to environmental conditions will be important for predicting their impact on plant communities and improving management programs, as well as how they relate to climate patterns.

## Figures and Tables

**Table 1:** The lm procedure (a two-way ANOVA linear model) of the effect of different factors on three components of cardinal temperature.

Cardinal Temperature	Parameter	Df	Sum_Squares	Mean_Square	*p*-Value
	Origin	1	416.11	416.11	<0.001 **
	Life cycle	2	37.13	18.56	0.1
Base	Populations	45	703.29	15.63	0.063 ·
	Species	24	705.81	4.64	0.008 **
	Year	1	89.04	89.04	0.004 **
	Origin	1	276.15	276.15	0.003 **
	Life cycle	2	106.78	53.39	0.098 *
Optimum	Populations	45	2064.29	45.87	0.058 ·
	Species	24	1132.21	47.18	0.058 ·
	Year	1	326.2	326.2	0.002 **
	Origin	1	320.81	320.81	<0.001 **
	Life cycle	2	215.53	107.76	0.009 **
Maximum	Populations	45	1594.85	35.44	0.054 ·
	Species	24	1361.32	56.72	0.012 *
	Year	1	40.92	40.92	0.115

**, *, and · are significant at 0.001, 0.01, and 0.05, respectively.

**Table 2:** The list of the species studied in this paper and their main characteristics.

Scientific Name	Family	Common Name	Life Cycle	Number of Ecotypes	Native Geographical Distribution
*Abutilon theophrasti* Medik.	Malvaceae	Velvetleaf	Annual	2	Central Asia to China
*Amaranthus blitoides* S.Wats.	Amaranthaceae	Prostrate pigweed	Annual	2	Central and E. Central U.S.A.
*Amaranthus retroflexus* L.	Amaranthaceae	Redroot pigweed	Annual	3	Mexico
*Amaranthus viridis* L.	Amaranthaceae	Slender amaranth	Annual	2	SE. Mexico to Tropical America
*Bassia scoparia* (L.) Beck	Amaranthaceae	Mexican fireweed	Annual	2	E. Europe to Temp. Asia
*Carthamus tinctorius* L.	Asteraceae	Safflower	Annual	3	Central and E. Türkiye to Iran
*Cleome viscosa* L.	Cleomaceae	Asian spiderflower	Annual	2	Tropical and Subtropical Old World
*Cucumis melo* L. subsp. *agrestis* var. *agrestis* (Naudin) Pangalo	Cucurbitaceae	Wild melon	Annual	2	Ethiopia to S. Africa, SW. Syria to Arabian Peninsula and Indian Subcontinent, New Guinea to N. and Central Australia.
*Cynanchum acutum* L.	Apocynaceae	Stranglewort	Perennial	3	Canary Islands (Lanzarote), Medit. to Siberia and N. China.
*Echinochloa crus-galli* (L.) P.Beauv.	Poaceae	Barnyard grass	Annual	2	S. and E. Europe to Asia, W., E., and S. Tropical Africa to S. Africa, Madagascar.
*Eruca sativa* (L.) Cav.	Brassicaceae	Rocket	Annual	2	Medit. to China and Arabian Peninsula
*Euphorbia maculata* L.	Euphorbiaceae	Spotted spurge	Annual	2	SE. Canada to Belize, Cuba, Bahamas
*Hordeum murinum* L.	Poaceae	Wall barley	Annual	2	Macaronesia, Europe, Medit. to Central Asia and W. Himalaya
*Hordeum vulgare* subsp. *spontaneum* (K.Koch) Asch. and Graebn.	Poaceae	Spontaneous barley	Annual	2	E. Medit. to Central Asia and China (Sichuan, Yunnan)
*Ipomoea nil* (L.) Roth	Convolvulaceae	Ivy morning glory	Annual	2	Tropical and Subtropical America
*Ipomoea purpurea* (L.) Roth	Convolvulaceae	Common morning glory	Annual	2	Tropical and Subtropical America
*Nigella sativa* L.	Ranunculaceae	Black cumin	Annual	3	Romania to W. and SW. Iran
*Papaver somniferum* L.	Papaveraceae	Opium poppy	Annual	2	Macaronesia, W. and Central Medit.
*Phalaris minor* Retz.	Poaceae	Little seed canary grass	Annual	3	Macaronesia, Medit. to Himalaya and Eritrea
*Plantago major* L.	Plantaginaceae	Greater plantain	Perennial	6	Temp. Eurasia to Arabian Peninsula, Macaronesia, N. and S. Africa
*Plantago ovata* Forssk.	Plantaginaceae	Blond plantain	Annual	3	SE. Spain, N. Africa to India and Somalia, SW. and S. Central U.S.A. to N. Mexico
*Polygonum aviculare* L.	Polygonaceae	Prostrate knotweed	Annual	2	Temp. Northern Hemisphere, Macaronesia to Eritrea
*Portulaca oleracea* L.	Portulacaceae	Common purslane	Annual	2	Macaronesia, Tropical Africa, Medit. to Pakistan and Arabian Peninsula.
*Prosopis farcta* (Banks and Sol.) J.F.Macbr.	Caesalpinioideae	Syrian mesquite	Perennial	4	N. Africa to Central Asia and India
*Rapistrum rugosum* (L.) All.	Brassicaceae	Turnipweed	Annual	2	Macaronesia, Medit. to Central Asia and Iran, NE. Tropical Africa to Arabian Peninsula
*Setaria italica* (L.) P. Beauvois	Poaceae	Foxtail millet	Annual	2	China
*Silybum marianum* (L.) Gaertn.	Asteraceae	Milk thistle	Biennial	5	Macaronesia, Medit. to Central Asia and India, Ethiopia
*Sinapis arvensis* L.	Brassicaceae	Wild mustard	Annual	3	Temp. Eurasia, N. Africa to Arabian Peninsula
*Sisymbrium irio* L.	Brassicaceae	London rocket	Annual	2	Europe to N. China and Himalaya, Sahara to N. and NE. Tropical Africa, Arabian Peninsula
*Trigonella foenum graecum* L.	Fabaceae	Fenugreek	Perennial	7	Iraq to N. Pakistan
*Xanthium strumarium* L.	Asteraceae	Rough cocklebur	Annual	3	S. Central and S. Europe to China and Indochina, Taiwan, NW. Africa

## Data Availability

The original contributions presented in this study are included in the Supplementary Materials; further inquiries can be directed to the corresponding author.

## Supplementary Materials

The following supporting information can be downloaded at https://www.mdpi.com/article/10.3390/plants13202848/s1.

## References

[1] Dubey P.S., Mall L.P. (1972). Ecology of germination of weed seeds. Oecologia.

[2] Rajjou L., Duval M., Gallardo K., Catusse J., Bally J., Job C., Job D. (2012). Seed germination and vigor. Annu. Rev. Plant Biol..

[3] Gioria M., Pyšek P. (2017). Early bird catches the worm: Germination as a critical step in plant invasion. Biol. Invasions.

[4] Kader M.A., Jutzi S.C. (2004). Effects of thermal and salt treatments during imbibition on germination and seedling growth of sorghum at 42/19 °C. J. Agron. Crop Sci..

[5] Schlaepfer D.R., Glättli M., Fischer M., van Kleunen M. (2010). A multi-species experiment in their native range indicates pre-adaptation of invasive alien plant species. New Phytol..

[6] Hao J.-H., Lv S.-S., Bhattacharya S., Fu J.-G. (2017). Germination response of four alien congeneric Amaranthus species to environmental factors. PLoS ONE.

[7] Ozaslan C., Farooq S., Onen H., Ozcan S., Bukun B., Gunal H. (2017). Germination biology of two invasive *Physalis* species and implications for their management in arid and semi-arid regions. Sci. Rep..

[8] Šerá B., Žarnovičan H., Hodálová I., Litavský J. (2024). Reproductive capacity and seed germination after various storage of the invasive alien plant *Amorpha fruticosa* L.—A case study from Bratislava. Biologia.

[9] Baker H.G. (1974). The evolution of weeds. Annu. Rev. Ecol. Evol. Syst..

[10] Zhang Q., Wang Y., Weng Z., Chen G., Peng C. (2024). Adaptation of the invasive plant *Sphagneticola trilobata* (L.) Pruski to drought stress. Plants.

[11] Wang C., Liu Y., Li C., Li Y., Du D. (2024). The invasive plant *Amaranthus spinosus* L. exhibits a stronger sesistance to drought than the native plant *A. tricolor* L. under co-cultivation conditions when treated with light drought. Plants.

[12] Hejda M., Pyšek P., Jarošík V. (2009). Impact of invasive plants on the species richness, diversity and composition of invaded communities. J. Ecol..

[13] Parepa M., Fischer M., Bossdorf O. (2013). Environmental variability promotes plant invasion. Nat. Commun..

[14] Hunter D.M., DeBerry D.A. (2023). Environmental drivers of plant invasion in wetland mitigation. Wetlands.

[15] Holm L., Doll J., Holm E., Pancho J., Herberger J. (1977). World Weeds: Natural Histories and Distribution.

[16] Huston M.A. (2004). Management strategies for plant invasions: Manipulating productivity, disturbance, and competition. Divers. Distrib..

[17] Johnson L.R., Handel S.N. (2019). Management intensity steers the long-term fate of ecological restoration in urban woodlands. Urban For. Urban Green..

[18] Kumschick S., Gaertner M., Vilà M., Essl F., Jeschke J.M., Pyšek P., Ricciardi A., Bacher S., Blackburn T.M., Dick J.T.A. (2014). Ecological impacts of alien species: Quantification, scope, caveats, and recommendations. BioScience.

[19] Sohrabi S., Gherekhloo J., Zand E., Nezamabadi N. (2023). The necessity of monitoring and assessing alien plants in Iran. Iran Nat..

[20] Soltani E., Baskin C.C., Gonzalez-Andujar J.L. (2022). An overview of environmental cues that affect germination of nondormant seeds. Seeds.

[21] Yan W., Hunt L.A. (1999). An equation for modelling the temperature response of plants using only the cardinal temperatures. Ann. Bot..

[22] Phartyal S.S., Thapliyal R.C., Nayal J.S., Rawat M.M.S., Joshi G. (2003). The influences of temperatures on seed germination rate in Himalayan elm (*Ulmus wallichiana*). Seed Sci. Technol..

[23] Hatfield J.L., Prueger J.H. (2015). Temperature extremes: Effect on plant growth and development. Weather Clim. Extrem..

[24] Garcia-Huidobro J., Monteith J.L., Squire G.R. (1982). Time, temperature and germination of pearl millet (*Pennisetum typhoides* S. & H.): I. Constant temperature. J. Exp. Bot..

[25] Gherekhloo J., Sohrabi S., Ansari O., Bagherani N., Prado R.D. (2023). Study of dormancy breaking and factors affecting germination of *Parapholis incurva* (L.) C.E.Hubb. as annual grass. Taiwania.

[26] Andreucci M.P., Moot D.J., Black A.D., Sedcole R. (2016). A comparison of cardinal temperatures estimated by linear and nonlinear models for germination and bulb growth of forage brassicas. Eur. J. Agron..

[27] Torabi B., Attarzadeh M., Soltani A. (2013). Germination response to temperature in different safflower (*Carthamus tinctorius*) cultivars. Seed Technol..

[28] Elahifard E., Derakhshan A. (2018). Asian spiderflower (*Cleome viscosa*) germination ecology in southern Iran. Weed Biol. Manag..

[29] Mijani S., Rastgoo M., Ghanbari A., Nassiri Mahallati M., González-Andújar J.L. (2021). Development of a new thermal time model for describing tuber sprouting of purple nutsedge (*Cyperus rotundus* L.). Weed Res..

[30] Taheri M., Gherekhloo J., Sohrabi S., Siahmarguee A., Hassanpour-bourkheili S. (2024). Sea barley (*Hordeum Marinum*) seed germination ecology and seedling emergence. Acta Bot. Hung..

[31] Sohrabi Kertabad S., Rashed Mohassel M.H., Nasiri Mahalati M., Gherekhloo J. (2013). Some biological aspects of the weed Lesser celandine (*Ranunculus ficaria*). Planta Daninha.

[32] Solouki H., Kafi M., Nabati J., Ahmadi-Lahijani M.J., Nezami A., Ahmady R.S. (2022). Quantifying cardinal temperatures of fenugreek (*Trigonella foenum graecum* L.) ecotypes using non-linear regression models. J. Appl. Res. Med. Aromat. Plants.

[33] Trotta G., Vuerich M., Petrussa E., Hay F.R., Assolari S., Boscutti F. (2023). Germination performance of alien and native species could shape community assembly of temperate grasslands under different temperature scenarios. Plant Ecol..

[34] Gioria M., Pyšek P., Osborne B.A. (2016). Timing is everything: Does early and late germination favor invasions by herbaceous alien plants?. J. Plant Ecol..

[35] Nešić M., Obratov-Petković D., Skočajić D., Bjedov I., Čule N. (2022). Factors affecting seed germination of the invasive species *Symphyotrichum lanceolatum* and their implication for invasion success. Plants.

[36] Maleki K., Soltani E., Seal C.E., Colville L., Pritchard H.W., Lamichhane J.R. (2024). The seed germination spectrum of 486 plant species: A global meta-regression and phylogenetic pattern in relation to temperature and water potential. Agric. For. Meteorol..

[37] Donohue K., Rubio de Casas R., Burghardt L., Kovach K., Willis C.G. (2010). Germination, postgermination adaptation, and species ecological ranges. Annu. Rev. Ecol. Evol. Syst..

[38] Colautti R.I., Lau J.A. (2015). Contemporary evolution during invasion: Evidence for differentiation, natural selection, and local adaptation. Mol. Ecol..

[39] Dlugosch K.M., Anderson S.R., Braasch J., Cang F.A., Gillette H.D. (2015). The devil is in the details: Genetic variation in introduced populations and its contributions to invasion. Mol. Ecol..

[40] Lu J.J., Tan D.Y., Baskin C.C., Baskin J.M. (2016). Effects of germination season on life history traits and on transgenerational plasticity in seed dormancy in a cold desert annual. Sci. Rep..

[41] Moyano J. (2023). Origins of successful invasions. Nat. Ecol. Evol..

[42] Tardieu F. (2013). Plant response to environmental conditions: Assessing potential production, water demand, and negative effects of water deficit. Front. Physiol..

[43] Gray S.B., Brady S.M. (2016). Plant developmental responses to climate change. Dev. Biol..

[44] Afuye G.A., Kalumba A.M., Orimoloye I.R. (2021). Characterisation of vegetation response to climate change: A review. Sustainability.

[45] Kiani M., Alahdadi I., Soltani E., Benakashani F. (2021). Changes of seed quality and germination of some black cumin ecotypes (*Nigella sativa* L.) during development and maturity. J. Plant. Prod. Res..

[46] Peco B., Rico L., Azcárate F.M. (2009). Seed size and response to rainfall patterns in annual grasslands: 16 years of permanent plot data. J. Veg. Sci..

[47] Kuniyal C.P., Purohit V., Butola J.S., Sundriyal R.C. (2013). Seed size correlates seedling emergence in *Terminalia bellerica*. S. Afr. J. Bot..

[48] Martins A.A., Opedal Ø.H., Armbruster W.S., Pélabon C. (2019). Rainfall seasonality predicts the germination behavior of a tropical dry-forest vine. Ecol. Evol..

[49] Münzbergová Z., Plačková I. (2010). Seed mass and population characteristics interact to determine performance of Scorzonera hispanica under common garden conditions. Flora Morphol. Distrib. Funct. Ecol. Plants.

[50] Mickelbart M.V., Hasegawa P.M., Bailey-Serres J. (2015). Genetic mechanisms of abiotic stress tolerance that translate to crop yield stability. Nat. Rev. Genet..

[51] Skálová H., Moravcová L., Pyšek P. (2011). Germination dynamics and seedling frost resistance of invasive and native Impatiens species reflect local climatic conditions. Perspect. Plant Ecol. Evol. Syst..

[52] Hulme P.E., Bernard-Verdier M. (2018). Comparing traits of native and alien plants: Can we do better?. Funct. Ecol..

[53] Haeuser E., Dawson W., van Kleunen M. (2019). Introduced garden plants are strong competitors of native and alien residents under simulated climate change. J. Ecol..

[54] Sohrabi S., Vilà M., Zand E., Gherekhloo J., Hassanpour-bourkheili S. (2023). Alien plants of Iran: Impacts, distribution and managements. Biol. Invasions.

[55] Dickson T.L., Hopwood J.L., Wilsey B.J. (2012). Do priority effects benefit invasive plants more than native plants? An experiment with six grassland species. Biol. Invasions.

[56] Fridley J.D. (2012). Extended leaf phenology and the autumn niche in deciduous forest invasions. Nature.

[57] Wainwright C.E., Wolkovich E.M., Cleland E.E. (2012). Seasonal priority effects: Implications for invasion and restoration in a semi-arid system. J. Appl. Ecol..

[58] Verdú M., Traveset A. (2005). Early emergence enhances plant fitness: A phylogenetically controlled meta-analysis. Ecology.

[59] Roché C.T., Thill D.C., Shafii B. (2017). Reproductive phenology in yellow starthistle (*Centaurea solstitialis*). Weed Sci..

[60] Sun Y., Kaleibar B.P., Oveisi M., Müller-Schärer H. (2021). Addressing climate change: What can plant invasion science and weed science learn from each other?. Front. Agron..

[61] Soler J., Izquierdo J. (2024). The invasive *Ailanthus altissima*: A biology, ecology, and control review. Plants.

[62] Gonzalez-Andujar J.L. (2023). Integrated weed management: A shift towards more sustainable and holistic practices. Agronomy.

[63] Gentili R., Schaffner U., Martinoli A., Citterio S. (2021). Invasive alien species and biodiversity: Impacts and management. Biodiversity.

[64] Rask A.M., Kristoffersen P. (2007). A review of non-chemical weed control on hard surfaces. Weed Res..

[65] Udugamasuriyage D., Kahandawa G., Tennakoon K.U. (2024). Nonchemical aquatic weed control methods: Exploring the efficacy of UV-C radiation as a novel weed control tool. Plants.

[66] Bussière F., Cellier P. (1994). Modification of the soil temperature and water content regimes by a crop residue mulch: Experiment and modelling. Agric. For. Meteorol..

[67] Gonzalez-Andujar J.L., Chantre G.R., Morvillo C., Blanco A.M., Forcella F. (2016). Predicting field weed emergence with empirical models and soft computing techniques. Weed Res..

[68] Lakens D. (2013). Calculating and reporting effect sizes to facilitate cumulative science: A practical primer for *t*-tests and ANOVAs. Front. Psychol..

[69] Ben-Shachar M.S., Lüdecke D., Makowski D. (2020). Estimation of effect size indices and standardized parameters. J. Open Source Softw..

[70] Sun Y., Wang C., Chen H.Y.H., Ruan H. (2020). Response of plants to water stress: A meta-analysis. Front. Plant Sci..

[71] Khodapanah G., Gherekhloo J., Sohrabi S., Ghaderi-Far F., Golmohammadzadeh S. (2023). Phenological response patterns and productive ability of Fallopia convolvulus to weather variability in Iran. Braz. J. Agric. Sci..

[72] Ghaderi-Far F., Gorzin M. (2019). Applied Research in Seed Technology.

